# Slow H_2_S-Releasing Donors and 3D Printable Arrays Cellular Models in Osteo-Differentiation of Mesenchymal Stem Cells for Personalized Therapies

**DOI:** 10.3390/biom14111380

**Published:** 2024-10-30

**Authors:** Ilaria Arciero, Silvia Buonvino, Sonia Melino

**Affiliations:** 1 Department of Chemical Sciences and Technologies, University of Rome “Tor Vergata”, Via della Ricerca Scientifica, 00133 Rome, Italy; ilaria-29@libero.it; 2 Department of Experimental Medicine, University of Rome “Tor Vergata”, Via Montpellier 1, 00133 Rome, Italy; silvia.buonvino95@gmail.com

**Keywords:** bone matrix, osteoarthritis, IL 6, osteocalcin, osteoblast, actin, garlic

## Abstract

The effects of the hydrogen sulfide (H_2_S) slow-releasing donor, named GSGa, a glutathione-conjugate water-soluble garlic extract, on human mesenchymal stem cells (hMSCs) in both bidimensional (2D) and three-dimensional (3D) cultures were investigated, demonstrating increased expression of the antioxidant enzyme HO-1 and decreased expression of the pro-inflammatory cytokine interleukin-6 (IL-6). The administration of the H_2_S donor can therefore increase the expression of antioxidant enzymes, which may have potential therapeutic applications in osteoarthritis (OA). Moreover, GSGa was able to promote the osteogenic differentiation of bone marrow mesenchymal stem cells (BMSCs), but not of cardiac mesenchymal stem cells (cMSCs) in a 2D culture system. This result highlights the varying sensitivity of hMSCs to the H_2_S donor GSGa, suggesting that the induction of osteogenic differentiation in stem cells by chemical factors is dependent on the tissue of origin. Additionally, a 3D-printable mesenchymal stem cells–bone matrix array (MSCBM), designed to closely mimic the stiffness of bone tissue, was developed to serve as a versatile tool for evaluating the effects of drugs and stem cells on bone repair in chronic diseases, such as OA. We demonstrated that the osteogenic differentiation process in cMSCs can be induced just by simulating bone stiffness in a 3D system. The expression of osteocalcin, RUNX2, and antioxidant enzymes was also assessed after treating MSCs with GSGa and/or increasing the stiffness of the culture environment. The printability of the array may enable better customization of the cavities, enabling an accurate replication of real bone defects. This could optimize the BM array to mimic bone defects not only in terms of stiffness, but also in terms of shape. This culture system may enable a rapid screening of antioxidant and anti-inflammatory compounds, facilitating a more personalized approach to regenerative therapy.

## 1. Introduction

H_2_S donors have acquired significant therapeutic potential for several diseases, such as cardiovascular [1,2,3], neurodegenerative [4,5,6,7], and gastrointestinal pathologies [8,9,10]. An increase in the endogenous production of hydrogen sulfide (H_2_S) has been observed in the differentiation process of mesenchymal stem cells in chondrocytes due to the overexpression of the enzymes involved in its synthesis [11]. Moreover, exogenous H_2_S can induce the up-regulation of enzymes involved in the synthesis of antioxidant molecules such as glutathione [12], which also regulates H_2_S production and stability. The anti-inflammatory and tissue repair properties of H_2_S have increased interest in the therapeutic potential of this *gasotransmitter* in arthritis. Osteoarthritis (OA) is a chronic disease characterized by inflammatory and degenerative processes, leading to the loss of cartilage and bone. It is characterized by severe oxidative stress and an acute immune response, with high expression of pro-inflammatory cytokines (such as interleukin-6, IL-6, and interleukin-1, and IL-1). The effect of H_2_S on joint inflammation has been investigated in recent years and primarily due to its anti-inflammatory properties. Recently, the role of endogenous H_2_S in regulating bone tissue development and function has gained attention [13,14,15]. Moreover, a strong correlation between OA progression and lower levels of H_2_S and/or the down-regulation of H_2_S production enzymes (3-mercaptopyruvate sulfurtransferase (3-MST), cystathionine gamma-lyase (CSE) and cystathionine beta-synthase (CBS) and) in the cartilage of OA patients has been established [11,16,17,18,19]. Several studies have demonstrated a protective effect of restoring endogenous H_2_S biosynthesis and treating damaged cartilage in OA patients with H_2_S donors. These studies show that supplementing H_2_S with slow H_2_S-releasing agents can efficiently limit inflammation, oxidative stress, and pain responses in the progression of OA [14,20,21,22,23]. This effect appears to be mediated by metalloproteinases inhibition of the matrix and the production of extracellular matrix proteins induced by H_2_S, which counteracted the catabolic effect of IL-1β [24,25]. Additionally, H_2_S is also able to reduce IL-1β-induced expression of IL-6, IL-8, MMP-2, and MMP-14 in OA fibroblast-like synoviocytes (FLS) [26]. The beneficial effect of H_2_S appears to be dose-dependent [27,28,29].

It is highly likely that the anti-inflammatory role of H_2_S is due to the inhibition of the transcription factor NF-κB, which plays a key role in the pathogenesis of inflammatory arthritis [30,31]. Furthermore, H_2_S can induce the synthesis of the anti-inflammatory cytokine IL-10 [32]. The inhibition of NF-κB activity by H_2_S reduces the production of pro-inflammatory cytokines, mediating the anti-inflammatory effect of the gas in an animal model of sepsis [33] and potentially in joint inflammation as well. Moreover, NF-κB is also a key factor in the differentiation and maturation of osteoclasts, which are responsible for bone erosions in arthritis [11].

Non-steroidal anti-inflammatory drugs (NSAIDs) have been conjugated with H_2_S [14,22] for slow H_2_S release into the target tissues. Interestingly, H_2_S conjugated to diclofenac inhibited osteoclastogenesis and mature osteoclasts, thus preventing osteolysis in an animal model of breast cancer metastasis. The inhibitory effect was demonstrated to be dependent on the IKK/NF-κB pathway [34,35]. H_2_S has a pleiotropic anti-inflammatory profile and may potentially act on bone erosion, the main long-term target in the treatment of arthritis. Several studies also highlight the therapeutic potential of garlic-derived H_2_S donors for the treatment of inflammatory arthritis; however, more in vitro and in vivo studies are needed to confirm the efficacy of these natural compounds, and their potentiality in inflammatory arthritis [22,36].

Currently, a surgical approach is most commonly used in advanced cases; however, this approach is not always suitable for elderly patients or those with mild joint damage. The regeneration of damaged tissue through human mesenchymal stem cells (hMSCs) and the possibility of inducing tissue repair in a damaged joint, combined with the anti-inflammatory effect of H_2_S, represents a very promising potential new approach. Sponge H_2_S-releasing silk fibroin (SF) [37,38] results in a scaffold with mechanical properties that match those of bone tissue and can induce a significant increase in the expression of osteogenic genes and in differentiation to mature osteoblasts after three weeks in a 3D culture model of hMSCs [37]. Nowadays, the use of H_2_S-functionalized or non-functionalized scaffolds, with or without cell delivery, is paving the way for a completely new therapeutic approach for treating different pathologies, including arthritis, as well as in tissue repair and regeneration [39,40,41,42]. In this study, we combined injectable hydrogel scaffolds with H_2_S donors and hMSCs to assess in vitro, the effects of H_2_S and stem cell administration using 3D cellular models for future potential clinical applications in OA. Three-dimensional cell-culture microsystems could be useful for mimicking the in vivo extracellular environment and studying how the tridimensionality and physical properties influence the differentiation of stem cells into different cell types [39,43,44].

In this work, we propose the development of 3D-printed arrays based on radiographic analyses, using a bone matrix (BM) ink capable of mimicking bone stiffness to better simulate the patient’s physiological bone tissue environment in terms of rheology, geometry and anisotropy. The array produced here provides a more reliable platform for in vitro testing of the effects of stem cells and drugs treatments before the administration to the patient and could potentially serve as a device for developing personalized therapies. The effects of both the stiffness of the cellular environment and a slow H_2_S donor, named GSGa, a glutathione-conjugate water-soluble garlic extract, [45,46] on the differentiation of two different mesenchymal stem cells lines derived from bone marrow (bone marrow mesenchymal stem cells (BMSCs)) and cardiac tissue (cardiac mesenchymal stem cells (cMSCs)) were investigated. The expression of osteocalcin, RUNX2 and antioxidant enzymes was assessed after treating MSCs with GSGa and/or increasing the stiffness of the cellular microenvironment. Furthermore, the production of the 3D-printable mesenchymal stem cells–bone matrix (MSCBM) array made it possible to demonstrate that the osteo-differentiation process of cMSCs can be induced by simulating bone stiffness, while H_2_S-donor administration can increase the expression of antioxidative enzymes, potentially leading to beneficial effects and therapeutical applications in OA.

## 2. Materials and Methods

### 2.1. Garlic Water-Soluble Extract Production from Allium sativum L.

GSGa was produced as described in our previous studies [45,46]. Briefly, a solution of 100 mM of reduced glutathione (GSH) in 50 mM Tris-HCl buffer, pH 7.5, was used to resuspend crushed garlic cloves (5 g). The resuspension and crushing were performed at low temperature in presence of liquid N_2._ The suspension was centrifuged and the water-soluble fraction was filtered (using 0.45 µm). The fraction GSGa was stored at −20 °C and analyzed by RP-HPLC, using mod. LC-10AVP (Shimadzu, Milan, Italy), equipped with a UV detector (Shimadzu, Milan, Italy) and a C_18_ column (150 mm × 4.6 mm, 5 μm, CPS Analitica, Rome, Italy). The solvent B gradient used for the chromatography was: 0–5 min, 0%; 5–55 min, 60%; 55–60 min, 60% and 65–85 min 90%, where 0.1% trifluoracetic acid was the solvent A and 80% CH_3_CN, 0.1% trifluoracetic acid was the solvent B. The elution was monitored at 220 nm and the concentration in mg/mL dry weight of GSGa was obtained by lyophilization of 100 µL.

### 2.2. H_2_S Release Assay and Cell Cultures Treatment with GSGa

The methylene blue (MB) assay was used in order to evaluate the H_2_S production and release by GSGa, as previously described [45,46]. Briefly, the extract with 1 mM dithiothreitol (DTT) in 50 mM Tris HCl, pH 7.4 (150 μL final volume) was incubated at 37 °C on a shaker for 30 min. Then, 20 μL of solution I (20 mM N′,N′-dimethyl-p-phenylene-diamine-dihydrochloride in 7.2 M HCl) and 20 μL of solution II (30 mM FeCl_3_ in 1.2 M HCl) were added after the incubation to each solution and after gentle mixing of the solutions for 10 min at room temperature the absorbance was measured at 670 nm. Na_2_S was used to elaborate a standard curve and the H_2_S release from GSGa was evaluated [45,46].

The treatments of 2D cMSC and BMSC (cell density of 5 × 10^3^ cells/cm^2^) and of 3D cMSC cultures (cMSCPSFs and cMSCBMs) (cell density of 5 × 10^3^ cells/µL) were performed adding in the cell culture medium, after 3 h of cell culture, 680 µg/mL of GSGa releasing 17 μM of H_2_S. Effects of GSGa were evaluated after 3 days of treatment.

### 2.3. 2D Cell Cultures and Hydrogel-Based Cell Microspheres Production

Cell studies were performed on bone marrow human mesenchymal stem cells BMSCs (Gibco, StemPro^®^, Life Technologies, Segrate, Italy), Sca-1^+^ Lin^−^ cardiac human mesenchymal stem cell line (cMSCs) and normal human dermal fibroblasts (NHDFs) (Lonza, Basel, Switzerland). The cMSC cell line was derived by cells extracted by auricular biopsies made during coronary artery bypass surgery from patients after signing a written consent form by Di Nardo et al., as previously described [39,46,47]. Cells were cultured in Dulbecco’s modified eagle medium (DMEM) high glucose (Gibco, Thermo Fisher Scientific, Milan, Italy), containing 10% of fetal bovine serum (FBS) (*v*/*v*) (Gibco, Thermo Fisher Scientific, Milan, Italy), 1% of penicillin-streptomycin (*w*/*v*) (Sigma-Aldrich, Mailand, Italy), 2 mM L-glutamine solution (Gibco, Thermo Fisher Scientific, Milan, Italy).

Hydrogel-based microspheres were produced by resuspending cMSCs or NHDFs in PEG-silk fibroin hydrogel (PSF) precursor solution obtained according to a published protocol [39,48]. Briefly, PEG-silk fibroin was produced by purification and PEGylation of the silk fibroin from *Bombix mori* cocoon [39] and, then, cells were resuspended at a density of 5 × 10^3^ cells/μL in PSF precursor solution (70 µL of PEG-silk fibroin 6 mg/mL fibroin, 30 μL of 30% *w*/*v* PEGDa 10 kDa, 1% *v*/*v* of a photoinitiator stock solution containing 10% *w*/*v* Irgacure^®^ 2959 (Ciba Specialty Chemicals, Basel, Switzerland) in 70% ethanol, for a total volume of 100 μL). Drops of 10 μL of the solution were put on a nanostructured super-hydrophobic surface of polydimethylsiloxane (PDMS) fabricated using the procedure described by Patent LDO0252 [49] (see Figure S1) and photopolymerized by exposure under UV light (365 nm, 4–5 mW cm^–2^) for 2 min. An extensive characterization of PSF hydrogel was performed in our previous studies [39,48]. Rheometric analysis of the PSF hydrogel was carried out previously [48], estimating a shear storage modulus (G’) of 13.4 kPa (Figure S2).

### 2.4. BM Array Production and 3D Cell Culturing

The 3D printable BM array has been created using BM ink (Stratasys, Minneapolis, MN, USA) by photopolymerization under UV light (365 nm, 4–5 mW cm^–2^) for 1 min inside a circular mold. The array cavities were obtained exploiting the hydrophobicity of the material by placing water drops of 10 µL before polymerization. cMSCs or NHDFs (at cell density of 5 × 10^3^ cells/µL) were embedded in PSF hydrogel spheres, photopolymerized directly into the cavities of the BM array to create 3D cell-BM cultures. The CAD drawing of the BM array, for printing the array using Stratasys J750 Digital Anatomy 3D Printer, was created using Blender Software (Blender, version 3.3, Amsterdam, The Netherlands).

### 2.5. Cell Viability Assay

The cell viability of cMSCs was assessed at day 0 and after 7 and 14 days of cell growth in BM array by WST-1(4-[3-(4-lodophenyl)-2-(4-nitrophenyl)-2H-5-tetrazolium]-1,3-benzene disulfonate (Cell Proliferation Reagent WST-1, Roche, Mannheim, Germany) assay [50]. The medium was replaced with fresh DMEM high glucose without phenol-red (GIBCO, Italy) containing tetrazolium salt WST-1 (5% *v*/*v*). cMSCBMs were incubated for 4 h at 37 °C, 5% CO_2_ and the absorbance of the medium was evaluated using a microplate reader at a wavelength of 450 nm.

### 2.6. Alizarin Red Staining and Alcian Blue Staining

Alizarin Red staining to detect calcium rich deposits was performed on stem cells and on cell culture medium after 3 or 5 days of cell growth. The culture medium was centrifuged and washed twice with H_2_O_dd_, stained with Alizarin Red solution pH 4.1 (Sigma-Aldrich, Italy) for 1 min and then centrifuged again; the staining of the precipitate was assessed by optical microscopy. Alizarin Red staining was also performed on cMSCs seeded on culture plate at day 3 of cell growth after fixing the cells with 4% paraformaldehyde (PFA), staining with Alizarin Red solution for 10 min and washing with H_2_O_dd_, according to the specific protocol. After 3 weeks of cell growth, 3D cMSCBM samples were stained by Alizarin Red following the same protocol described above, and by Alcian Blue staining to detect chondrogenic differentiation. Alcian Blue staining was performed fixing the spheres with 4% PFA, staining with Alcian Blue solution pH 2.5, for 10 min and washing with H_2_O_dd_. The Alizarin Red and Alcian Blue staining were assessed by optical microscopy using Zeiss microscope (Primovert, Zeiss, Oberkochen, Germany). The quantitative analyses of the Alizarin Red S staining were performed at a wavelength of 595 nm using a microplate reader (iMark BIO-RAD, Hercules, CA, USA).

### 2.7. Optical and Fluorescence Microscopy Analyses

Cell nuclei staining of live cells with Hoechst 33342 (Sigma-Aldrich, Italy) of cMSCs or NHDFs in BM array was performed after 1 week or 1 day of cell growth, respectively.

cMSCPSF spheres and cMSCBM array were analyzed by immunofluorescence staining. At selected times (3 and 30 days), samples were fixed with 4% PFA in PBS for 30 min at room temperature, then permeabilized with 0.3% Triton X-100 for 5 min and maintained in blocking buffer (10% *v*/*v* FBS, 0.1% *v*/*v* Triton X-100, and 1% *w*/*v* glycine in PBS) overnight at 4 °C. 3D samples were incubated overnight at 4 °C with 1:200 *v*/*v* Ab-α-smooth muscle actin (α-SMA) (Thermo Fisher Scientific, Invitrogen, Waltham, MA, USA) or Ab-α actinin (Sigma-Aldrich, Italy) or Ab-osteocalcin (Thermo Fisher Scientific, Invitrogen, USA) in PBS with 1% albumin with 20 mM Gly solution, followed by 4 h of incubation with the appropriate 1:200 *v*/*v* Alexa-fluorochrome-conjugated secondary antibody (488 nm green or 546 nm red) in 20 mM Gly-PBS at room temperature (Thermo Fisher Scientific, Invitrogen, USA). Cell nuclei were stained with Hoechst 33342 (Sigma-Aldrich, Italy). Fluorescence micrographs were captured using a Zeiss Axio Observer 7 microscope and confocal microscopy was performed using a Stellaris Leica microscope platform. The densitometric analysis of the immunofluorescence micrographs was performed using Image J software. The fluorescence intensity of osteocalcin was expressed in corrected total cell fluorescence (CTCF) that was calculated using the following equation:

CTCF = integrated density − (area of selected cell × mean fluorescence of background readings).

### 2.8. Western Blot Analysis

Proteins were extracted from cells grown in 2D and 3D culture systems using 50 μL of sample buffer for SDS-PAGE. Samples were vortex and boiled for 5 min and centrifuged for 5 min at 10,000 rpm and stored at −20 °C. SDS-PAGE of cell extracts was performed using 12% polyacrylamide gel, while for electro-blotting PVDF membranes (Sigma–Aldrich, Italy) were used. The membranes were then blocked and probed overnight at 4 °C with primary monoclonal antibodies: Ab-RUNX2 mouse (Abcam, Cambridge, UK), Ab-IL-6 mouse (Thermo Fisher Scientific, Invitrogen, USA), Ab-HO-1 rabbit (Cell Signaling Technology, Danvers, WA, USA), Ab-osteocalcin mouse (Thermo Fisher Scientific, Invitrogen, USA), Ab-β-actin rabbit (Sigma-Aldrich, Italy). Immunoblots were next incubated with the secondary antibodies (dilution 1:3000) (Cell Signaling Technology, USA) for 4 h at room temperature. Immunoblot with Ab-vinculin mouse (Santa Cruz Biotechnology, USA), Ab-GAPDH rabbit (Sigma-Aldrich, Italy), and Ab-β-tubulin mouse (Sigma-Aldrich, Milan, Italy) were also probed for controlling the protein loading. Super Signal West Pico kit (Thermo Scientific, USA) was used for developing immunoblotting, followed by exposure to a Fluorchem Imaging system (Alpha Innotech Corporation-Analitica De Mori, Milan, Italy). The quantitative evaluation of protein expression for 3D cultures was obtained by 3–5 microspheres for each sample and is therefore also inherently mediated.

### 2.9. Statistical Analysis

All data (protein expression, cell viability, MB assay and Alizarin Red S assay) were obtained by three or more experimental independent biological replicates and were presented as the mean ± standard deviation (S.D.). The statistical analysis was performed using the t-test or the one-way ANOVA test followed by Dunnett’s multiple comparisons test. A *p*-value < 0.05 was considered statistically significant. The program used for the statistical analysis was GraphPad Prism version 8.0 for Windows (GraphPad Software, San Diego, CA, USA).

## 3. Results and Discussion

### 3.1. Effects of H_2_S Slow-Releasing Agents on the Osteo-Differentiation of MSCs and 3D Cultures

**Figure 1:**
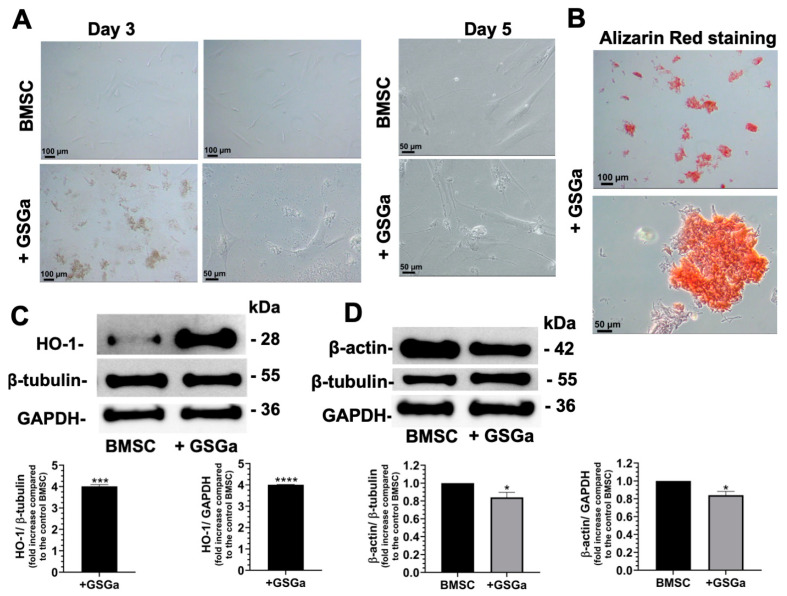
Effects of GSGa on BMSCs in 2D culture. (**A**) Optical micrographs of BMSCs after 3 and 5 days of cell growth in the presence and in the absence of GSGa (680 µg/mL releasing 17.0 µM of H_2_S) (+ GSGa); (**B**) Alizarin Red staining of the pellet of culture medium of BMSCs cultured for 3 days in the presence of GSGa; (**C**) Western blot analysis of the expression of HO-1 in BMSCs grown 5 days in the presence (+ GSGa) and in the absence of GSGa; (**D**) Western blot analysis of the β-actin expression in BMSCs cultured for 5 days in the absence and in the presence of GSGa. Experiments were performed as three biological replicates; Error bars indicate S.D. * *p* value ≤ 0.05, *** *p* value ≤ 0.0005, **** *p* value ≤ 0.0001. Scale bars are of 100 μm and 50 μm.

Firstly, the effects of GSGa on 2D cultures of BMSCs were evaluated. After three days of cell growth in the presence of GSGa (680 µg/mL), releasing 17.0 µM of H_2_S, optical microscopy of the treated samples showed the presence of Alizarin Red S (ARS)-positive deposits released by the cells in culture medium (Figure 1A), suggesting an initial commitment in the osteogenic differentiation of BMSCs (Figure 1B). The quantitative analyses of ARS staining are reported in the supplementary materials (Figure S3). These findings are in agreement with the role exerted by H_2_S in osteogenic differentiation of MSCs [13], where the endogenous production of H_2_S in BMSCs was found to regulate their self-renewal and osteogenic differentiation, while H_2_S deficiency led to a reduced ability of BMSCs to differentiate into osteoblast cells [51].

Moreover, a statistically significant increase in the heme oxygenase-1 (HO-1) expression was observed in treated BMSCs (Figure 1C), in agreement with data previously obtained on cMSCs [44,46]. HO-1 is a stress-inducible enzyme that provides protection against oxidative damage induced by ROS, with significant anti-inflammatory properties [52,53,54,55,56]. H_2_S release promotes the expression of HO-1 and other antioxidant enzymes, such as NAD(P)H dehydrogenase quinone 1 (NQO1) and glutathione-peroxidase 1 (GPx1) regulating the Nrf2-ARE (antioxidant response elements) pathway [46,57]. Indeed, H_2_S induces the dissociation between Nrf2 and Keap1 via Keap1 sulfhydration at the level of the Cys151 residue [57], allowing Nrf2 to translocate into the nucleus and bind to ARE, inducing antioxidant enzyme expression [14,44]. Therefore, the GSGa-mediated up-regulation of antioxidant enzymes can play a significant role in protection against oxidative stress and cellular damage which occur in osteoarthritis [14]. The activation of the cellular antioxidant system in stem cells led us to suggest the potential use of GSGa in osteoarthritis treatment.

Interestingly, we also found a reduction in the β-actin expression level after five days of treatment, in comparison with two normalization proteins (GAPDH and β-tubulin), as shown in Figure 1D. This reduction could be due to a change in gene expression or a proteasomal degradation of the actin protein, related to the early stages of cytoskeleton morphological remodeling during differentiation. This result may suggest that actin protein could be a marker in osteogenic differentiation. Some recent studies reported that β-actin expression can change in response to biochemical stimuli during cell growth and differentiation. In particular, loss of β-actin was found to lead to accelerated matrix mineralization, accompanied by enhanced formation of extracellular hydroxyapatite microcrystals and the induction of the expression of osteogenic biomarkers, such as osteocalcin or osteopontin [58]. The investigation into detailed effects of GSGa on the induction of calcium deposits release during the initial commitment of osteogenic differentiation is ongoing. Further studies are required to better identify the activation pathway induced by GSGa treatment in BMSCs.

**Figure 2:**
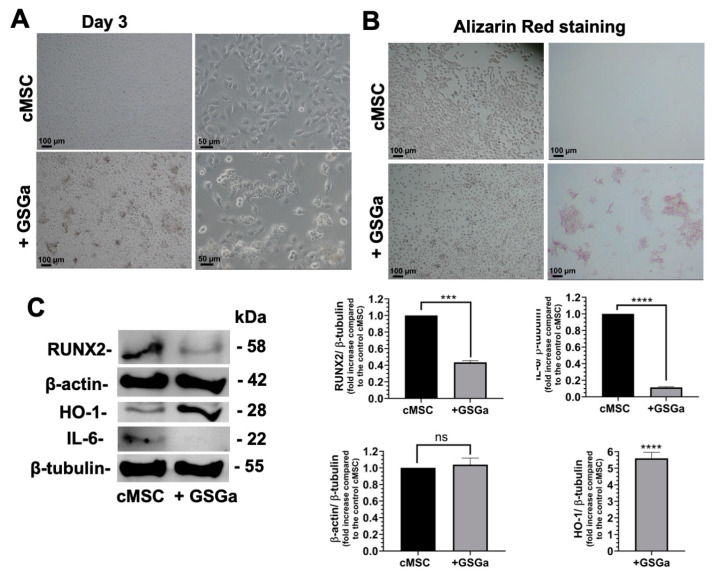
Effects of GSGa on cMSCs in 2D cultures. (**A**) Optical micrographs of cMSCs after 3 days of cell growth in the presence (+ GSGa) and in the absence (cMSCs) of GSGa (680 µg/mL releasing 17.0 µM of H_2_S); (**B**) Alizarin Red staining of cMSCs in the absence and in the presence of GSGa after 3 days of cell growth; (**C**) Western blot analysis of the expression of RUNX2, β-actin, IL-6 and HO-1 in cMSCs after 3 days of cell growth in the presence and in the absence of GSGa. Experiments were performed as three biological replicates. Error bars indicate S.D. ns = non-significant, *** *p* value ≤ 0.0005, **** *p* value ≤ 0.0001. Scale bars are of 100 µm and 50 µm.

The effects of GSGa were also assessed on cMSCs grown in 2D cultures, using the same concentration of GSGa. After three days of cell growth in the presence of GSGa, the substances released into the culture medium by the cells (Figure 2A) were only weakly positive to Alizarin Red staining (Figure 2B and Figure S3). The expression of RUNX2, a key transcription factor associated with early stages of osteogenesis (Figure 2C), was here evaluated in the cMSCs grown in the presence of GSGa; down-regulation and no significant changes in actin expression were observed. Therefore, these results show that osteogenic differentiation did not occur in 2D cultures of cMSCs after GSGa treatment, demonstrating that the effects of the H_2_S donor on stem cell differentiation are dependent on the type of mesenchymal stem cell line. However, a significant up-regulation of HO-1, together with the down-regulation of IL-6, were observed after treatment, as shown in Figure 2C. These results are in agreement with the observed ability of H_2_S donors to activate the Nrf2-ARE pathway and inhibit the expression of pro-inflammatory cytokines (such as IL-6), through the inhibition of NF-kB activity [14,44,59,60,61]. The role of NF-κB in the expression of pro-inflammatory genes including cytokines, chemokines, and adhesion molecules is well known [62,63,64] and the inhibition of the NF-kB pathway could have a relevant role in the therapeutic treatment of inflammatory diseases, such as osteoarthritis. Therefore, H_2_S donors are emerging as potential regulators of inflammation and antarthritic drugs [14,22,65,66].

**Figure 3:**
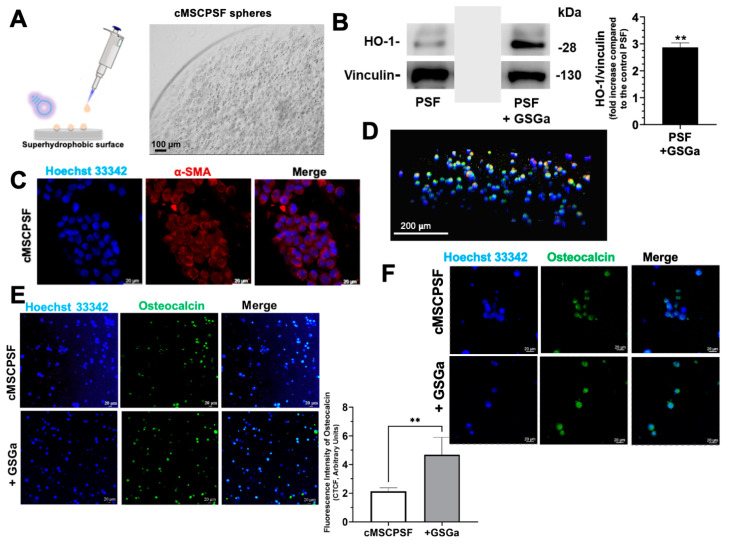
3D cMSCPSF cultures and effects of GSGa on the cells. (**A**) Schematic representation of the MSCPSF spheres production and micrograph of a representative sphere; (**B**) expression of HO-1 in cMSCPSF spheres (PSF_soft_, 4.5% of PEGDa w/v) (10 μL) embedded at cell density of 5 × 10^3^ cells/ µL after 3 days of cell culture in the absence (PSF as control) and in the presence (PSF +GSGa) of GSGa (680 µg/mL); (**C**) confocal micrographs of cMSCPSF spheres after 7 days of cell growth. Nuclei are in blue stained with Hoechst 33342 and α-SMA is in red stained using Ab-α-SMA-Alexa-Fluor 546 nm. Z stack are obtained by the overlapping of 52 slices; (**D**) 3D projection of cMSCPSF spheres after 7 days of cell growth stained with Ab-α-SMA-Alexa-Fluor 546 nm (red) and Ab-osteocalcin-Alexa-Fluor 488 nm (green); (**E**) confocal micrographs of the osteocalcin expression in cMSCPSF after 3 days of cell culture in the absence and in the presence of GSGa (Z-stack obtained by 137 and 132 slices, respectively) by immunostaining with Ab-osteocalcin-Alexa-Fluor 488 nm (in green); (**F**) confocal micrographs obtained with zoom 2.5, Z-stack obtained by 127 slices for cMSCPSF and 99 slices for + GSGa. Densitometric analysis of the immunofluorescence micrographs of MSCPSF cultured in the presence and in the absence of GSGa expressed as corrected total cell fluorescence (CTCF). Error bars indicate S.D. ** *p* value ≤ 0.01. Scale bars are of 20, 100 and 200 µm.

The effects of GSGa, at a concentration of 680 µg/mL, were also investigated using 3D cell cultures of cMSCs based on PSF hydrogel. PSF hydrogel is a photopolymerizable hydrogel that combines the bio-functionality of silk fibroin (SF) with the structural versatility of polyethylene-glycol-diacrylate (PEGDa). Furthermore, PSF shows tunable mechanical properties, including stiffness, that can be easily modulated by changing the concentration of PEGDa in the gel precursor solution. cMSCPSF spheroids (10 μL, at a cell density of 5 × 10^3^ cells/µL) were produced using PSF_soft_ (4.5% PEGDa *w*/*v*), characterized by a stiffness of ~5 kPa (Figure 3A). Increased expression of HO-1 in cMSCs cultured in PSF_soft_ after three days of GSGa treatment was observed (Figure 3B). At the same concentration of GSGa, the increase in HO-1 expression was lower compared to that observed in 2D cultures of cMSCs, probably due to a lower concentration and diffusion rate of the *gasotransmitter* in the 3D cellular system. Therefore, this result confirmed the importance of testing and optimizing the administration of the H_2_S slow-releasing compounds in 3D cellular models before the in vivo experiments.

Confocal micrographs in Figure 3 show the expression of both alpha-smooth muscle actin protein (α-SMA) and osteocalcin in the cMSCPSF system (Figure 3C–F). cMSCs grown for 1 week in PSF_soft_ showed good α-SMA expression (Figure 3C). However, the globular cell morphology prompted us to investigate whether the stiffness of the 3D PSF system could induce differentiation into cell types other than myocytes. Figure 3D shows the 3D projection of the merged micrographs obtained from immunostaining using Ab-α-SMA and Ab-osteocalcin, demonstrating the co-overexpression of the two proteins after 1 week of cell growth in PSF. Furthermore, we assessed osteocalcin expression and observed a good expression of this protein in the cMSCPSF system after only 3 days of culture (Figure 3E,F). These results are in agreement with a previous study in which it was demonstrated that stiff cultures can stimulate hMSCs to up-regulate the α-SMA expression in the early stage of the cell osteo-differentiation process [67]. Osteocalcin expression was significantly increased by GSGa treatment, as demonstrated by immunofluorescence confocal microscopy analyses (Figure 3E,F and Figure S4). These results suggested that GSGa in the presence of physical stimuli, such as stiffness, is able to further improve the osteogenic differentiation in cMSC 3D cell cultures.

### 3.2. Three-Dimensional Hydrogel-Based Array for Modelling the Disease: The Mini-Bone System

**Figure 4:**
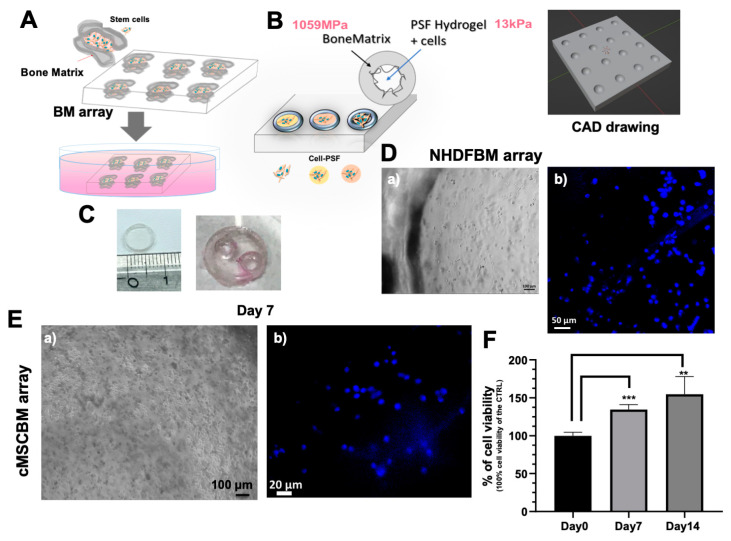
3D BM array design, production and assessment for cell growth. (**A**) Scheme of 3D printable BM array mimicking the bone section for the in vitro modelling of osteoarthritis; (**B**) representation of 3D PSF cell cultures inside the BM array and CAD drawing of the array; (**C**) digital images of BM mold and prototype array for cell culturing; (**D**) (a) brightfield and (b) fluorescence micrographs of NHDFBM (with PFS_stiff_) after 24 h of cell culture. Nuclei are in blue and were stained with Hoechst 33,342 (staining of live cells); (**E**) (a) brightfield and (b) fluorescence micrographs of cMSCBM array (with PSF_stiff_) after 7 days of cell growth, the nuclei are stained with Hoechst 33342 (staining of live cells); (**F**) WST-1 cell viability assay of cMSCBM at 0, 7 and 14 days of cell growth. Each result was obtained by three or four independent experiments. Error bar indicates S.D. ** *p* value ≤ 0.01, *** *p* value ≤ 0.0005. Scale bars are of 50, 100 and 20 μm.

In addition to the tridimensionality other physical factors, such as stiffness, the geometry and anisotropy of the section can be relevant stimuli in the induction of the physiological differentiation process of MSCs. Therefore, our aim was to produce 3D-printable bone matrix arrays (BM array) with the tunable MSCPSF system to better simulate the physiologic conditions in vitro and evaluate sites and times of the stem cell differentiation process. This would allow us to investigate the effects of drugs to identify the appropriate stem cells and drug administration for a personalized therapy in patients. Starting from imaging techniques, such as magnetic resonance imaging (MRI), customized bone defects could be reproduced in the BM array using 3D printing, to mimic the damaged bone section (Figure 4A).

To study the effects of bone tissue stiffness on stem cells, we developed a 3D cell-culture system array using BM ink for 3D printers. This ink is commonly used to create 3D-printed models of bone sections with realistic design and stiffness, making it useful for training, surgical planning, and the development of medical devices. The BM ink is characterized by a tensile modulus of 1059 ± 31 MPa. This is similar to the elastic modulus of human trabecular bone, which typically falls within the range of 1.3–7.8 GPa, while that of cortical bone ranges between 12 and 20 GPa [68,69]. However, tissues show different degrees of stiffness depending on the physio-pathological state. For instance, the stiffness of the subchondral bone plate in both osteoporosis and osteoarthritis patients is much lower than that in normal conditions [70]. This suggests that this ink is a suitable material for BM array development.

To optimize the production conditions of the BM array, a mold measuring 1 cm in diameter was produced using photopolymerization under UV light, as shown in Figure 4. Leveraging the hydrophobicity of the material, we produced semispherical cavities with water drops (10 µL) on the BM array during the photopolymerization process. The array was then sterilized using an autoclave, without any alteration in shape, dimensions and optical transparency, which is relevant for microscopic analyses (Figure 4). Figure 4B shows the CAD drawing designed for printing the array. Cell adhesion and proliferation on the BM array were also assessed using a cell metabolic assay. Cell adhesion and viability were reduced compared to those of cells seeded and grown on tissue culture plate (TCP) (see Figure S5A, Supplementary Materials), likely due to the high hydrophobicity and a cytotoxic effect of the BM ink. The cytotoxicity of the material was reduced via incubation of the BM array for one week with PBS and two days in cell culture medium. Cells cultured with pretreated BM showed a cell viability of 31.61% ± 3.41% higher compared to those cultured with un-treated BM (Supplementary Materials Figure S5B). Furthermore, cells were embedded into PSF hydrogel spheres through direct photopolymerization into the cavities of the pre-treated BM array (Figure 4B) and the MSCBM array was cultured. The use of hydrogels is particularly advantageous in the development of 3D culture systems because they can mimic the mechano–physical and biological properties of native tissues, thanks to the tunability of their physical–chemical properties. Herein, 3D hydrogel-based cell cultures were incorporated in the BM array to create a more complex and comprehensive device for the investigation of the biochemical and mechano–physical stimuli that drive MSC differentiation in the context of osteoarthritis disease. As a preliminary experiment to assess cell culturing in the BM array, normal human dermal fibroblasts (NHDFs) were embedded in PSF hydrogel with 9% *w*/*v* PEGDa (10 kDa) (PSF_stiff_) and photopolymerized directly within the array; good cell viability was observed using fluorescence microscopy with Hoechst staining of live cells (see Figure 4D). An increase in cell proliferation over 2 weeks was observed in the cMSCBM array (Figure 4F). In Figure 4E are shown brightfield micrographs of the cMSCBM array and the fluorescence micrograph of cells stained with Hoechst 33,342 (nuclei staining of live cells) performed after 1 week of cell growth. Fluorescence analysis confirmed the good cell viability and integrity of the nuclei.

### 3.3. Osteo-Differentiation in 3D Mini-Bone Systems

Moreover, we considered the physical factor of increased stiffness due to the presence of the BM array, which has a stiffness of 1059 MPa, in addition to the stiffness of the PSF hydrogel.

**Figure 5:**
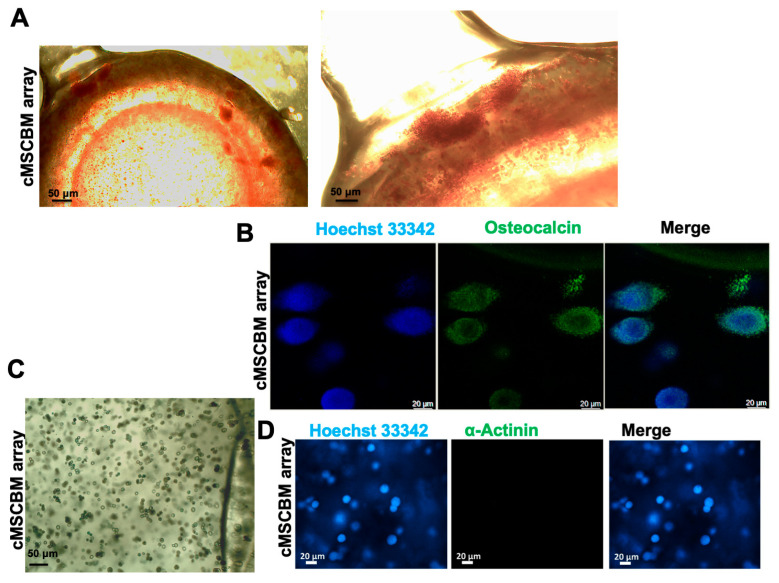
cMSC differentiation in MSCBM array. (**A**) Alizarin Red staining of cMSCBMs with PSF_stiff_ performed after 3 weeks of cell culture; (**B**) confocal micrographs of cMSCBMs after 30 days of cell culture, the osteocalcin expression is in green obtained by Ab-osteocalcin-Alexa-Fluor 488 nm (Z-stack by overlapping of 100 slices); (**C**) Alcian Blue staining of cMSCBM array (with PSF_stiff_) performed after 3 weeks of cell growth; (**D**) confocal micrographs of cMSCBM array after 3 weeks of cell culture, the α-actinin expression (in green) was evaluated using Ab-α-actinin-Alexa-Fluor 488 nm (Z-stack by overlapping of 100 slices). Nuclei are in blue by staining with Hoechst 33342. Scale bars are of 100, 50 and 20 μm.

**Figure 6:**
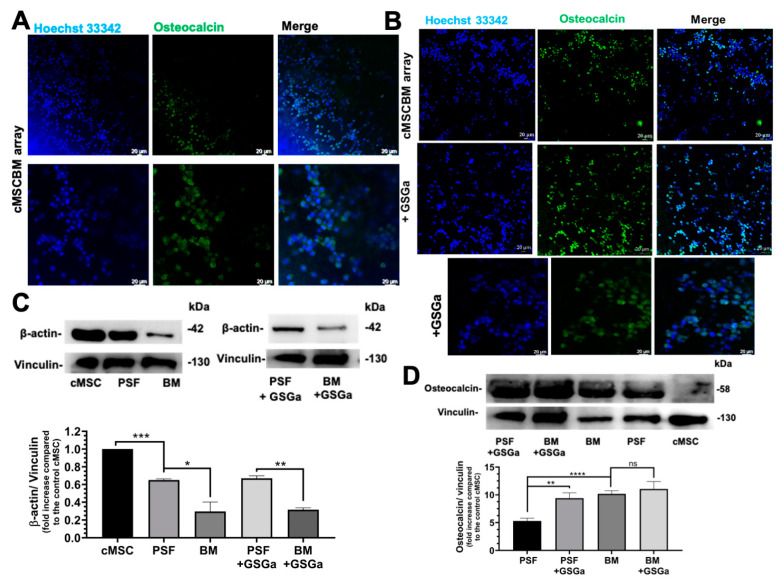
BM array promotes osteogenic differentiation of cMSCs. Confocal micrographs of cMSCBM array after 3 days of cell culture in the absence (**A**) and in the presence (**B**) of GSGa (+ GSGa) obtained using Z-stack by overlapping of 93 (A, BM array) and 78 (+ GSGa), 81 (B, BM array) and 76 slices (+ GSGa); the osteocalcin was stained in green, and the nuclei in blue were stained with Hoechst 33342. Expression of β-actin (**C**) and osteocalcin (**D**) by Western blotting analysis of cMSCs grown in 2D culture (cMSC), 3D cMSCPSF (PSF), cMSCBM array (BM) and in the presence of GSGa (PSF+GSGa) (BM+GSGa) after 3 days of cell growth. Error bars indicate S.D. ns = non-significant, * *p* value ≤ 0.05, ** *p* value ≤ 0.01, *** *p* value ≤ 0.0005, and ****p value ≤ 0.0001. Scale bars are of 20 μm.

In Figure 5 are shown the micrographs of the Alizarin Red assay (Figure 5A) of the MSCBM array after 3 weeks of culture. The presence of calcium deposits, stained in red, especially located at the edges of the well, demonstrates the osteo-differentiation of the cMSCs in the array, further confirmed by the presence of high expression of osteocalcin (Figure 5B). In agreement, the negative Alcian Blue staining (Figure 5C) and absence of the alpha-actinin (Figure 5D) demonstrated that the cells were not driven through either chondrocyte or muscle differentiation. In the MSCBM array, the cMSCs showed a higher osteocalcin expression in proximity to the BM matrix (Figure 6A). Moreover, Western blotting analysis demonstrated increased osteocalcin expression in this system with respect to MSCPSF spheres (Figure 6D), suggesting that the physical factor of the increased stiffness leads to a significant increase in osteogenic differentiation. A further significant reduction in the β-actin expression level in a stiffness-dependent manner was observed in the cMSCBM array with PSF_soft_ compared to both 2D cultures of cMSCs and cMSCPSF_soft_ spheres (Figure 6C). No significant differences in osteocalcin and β-actin expression were observed in the cMSCBM array in the presence of GSGa, as shown in confocal micrographs obtained by immunostaining and Western blot analysis (Figure 6B–D). This result suggests that in the 3D models that simulate the physiological rheology of the bone tissue, GSGa may be relevant for its anti-inflammatory effects, as it reduces IL-6 and ROS levels, without increasing the osteogenic differentiation, which is driven by the physical stimulus (physically induced differentiation (PiD)) in hMSCs derived from cardiac muscle.

The up-regulation of HO-1 and inhibition of the NF-κB pathway induced by H_2_S donors such as GSGa may lead to the inhibition of vascular cell adhesion molecule 1 (VCAM1) expression [71,72]. The increased production of VCAM1 and intracellular adhesion molecule 1 (ICAM1) in endothelial cells are initial events in the inflammation response linked to leukocyte recruitment and tissue infiltration at the site of inflammation. Therefore, the administration of H_2_S donors combined with hMSCs in osteoarthritis may promote a reduction in the inflammatory process and support the regeneration of bone tissue.

## 4. Conclusions

The effects of the slow-H_2_S releasing donor GSGa on hMSCs in both 2D and 3D cultures were investigated, demonstrating increased expression of the antioxidant enzyme HO-1 and decreased expression of the pro-inflammatory cytokine IL-6. Moreover, GSGa was able to promote the osteogenic differentiation of BMSCs but not that of cMSCs cultured in a 2D system. This result demonstrates the different sensitivity of the hMSCs to the H_2_S- donor GSGa and indicates that the induction of osteogenic differentiation of the stem cell by chemical factors is dependent on the tissue from which it derives. Our results are also in agreement with studies that demonstrate that MSCs, isolated from different tissues, retain their epigenetic memory and exhibit varying differentiation capabilities in vitro, depending on their tissue of origin [73]. The fate of the stem cells may be driven by the biochemical and mechano–physical properties of the 3D environment in which they are embedded [68,74,75,76,77,78].

A 3D printable BM array, with a tunable cell embedding PSF hydrogel, was here produced as a proof of concept for the production of a more complex personalized platform, using a commercial “*ink*”. PSF hydrogel may be injected and photopolymerized in situ representing a suitable candidate for regenerative medicine applications, also in osteoarthritis treatment. In addition, its properties make PSF a potential suitable ink for 3D bioprinting technology and future studies will allow to set up the optimal conditions for its printing. MSCBM array was used for assessing the differentiation of hMSCs in the presence and absence of a H_2_S donor and rheological stimuli. The effects of chemical and physical stimuli on osteogenic differentiation in the 3D-printable hMSCBM array were observed, demonstrating a relevant effect of the stiffness in the increase in osteocalcin expression in hMSCs, which could not be differentiated in a 2D culture system in the presence of our H_2_S- donor. Moreover, the increased stiffness in the MSCBM array led to a significant increase in osteogenic differentiation, compared to the 3D culture with PSF_soft_. These results are also in agreement with our previous studies, in which it was observed that cMSCs embedded into PSF hydrogel sponge-like scaffolds, with microspheres and lower stiffness, are able to express alpha-actinin and connexin-43 after three weeks of cell growth, characteristic of an initial differentiation into cardiac muscle [39]. Interestingly, here, a significant reduction in the β-actin expression levels in a stiffness-dependent manner was observed, which was related to the osteogenic differentiation process. Actin protein is essential for maintaining the structure of the cytoskeleton, which, in turn, plays significant role in stem cell differentiation [79]. Recently, a loss of β-actin and changes in its polymerization were observed related to accelerated matrix mineralization, enhanced formation of extracellular hydroxyapatite microcrystals, and the induction of the expression of osteogenic biomarkers, such as osteocalcin [58,80]. Therefore, the decrease in β-actin expression levels could be associated with osteogenesis, and is likely due to minor expression and/or degradation of this protein related to cytoskeleton remodeling, which occurs during osteo-differentiation.

The 3D printable MSCBM array, which closely mimics the stiffness of the bone tissue environment, may represent a versatile tool for assessing the effects of drugs and cells on the bone repair in chronic diseases, such as osteoarthritis, allowing for rapid drug screening of antioxidant and anti-inflammatory compounds for a more personalized regenerative therapy. The printability of the array could also enable better customization of the cavities to accurately replicate real bone defects, thereby optimizing the BM array to mimic bone defects in terms of both stiffness and shape.

## Data Availability

The authors declare that all data generated in this study are available within the article or the Supplementary Information. Other data related to this work are available from the corresponding authors upon request.

## Supplementary Materials

The following supporting information can be downloaded at: https://www.mdpi.com/article/10.3390/biom14111380/s1, Figure S1: Nanostructured superhydrophobic surface; Figure S2: Rheological analysis of PSF hydrogel; Figure S3: Quantitative analysis of the Alizarin Red staining of hMSCs after GSGa treatment; Figure S4: Osteocalcin expression in 3D MSCPSF grown in the presence of GSGa; Figure S5: Cell adhesion and viability on bone matrix; Figures S6–S9: Original Western Blotting of Figure 1, Figure 2, Figure 3 and Figure 6.
