# Supplementary material for: Slow H2S-Releasing Donors and 3D Printable Arrays Cellular Models in Osteo-Differentiation of Mesenchymal Stem Cells for Personalized Therapies
Source: Biomolecules. 2024 Oct 30;14(11):1380. doi: 10.3390/biom14111380 (PMC11592188; doi:10.3390/biom14111380)
Supplement: Supplementary file 1 [file biomolecules-14-01380-s001.zip › biomolecules-3250533-supplementary.pdf]

biomolecules

Supplementary Materials

Slow H_2S-Releasing Donors and 3D Printable Arrays Cellular Models in Osteo-Differentiation of Mesenchymal Stem Cells for Personalized Therapies

Ilaria Arciero, Silvia Buonvino and Sonia Melino

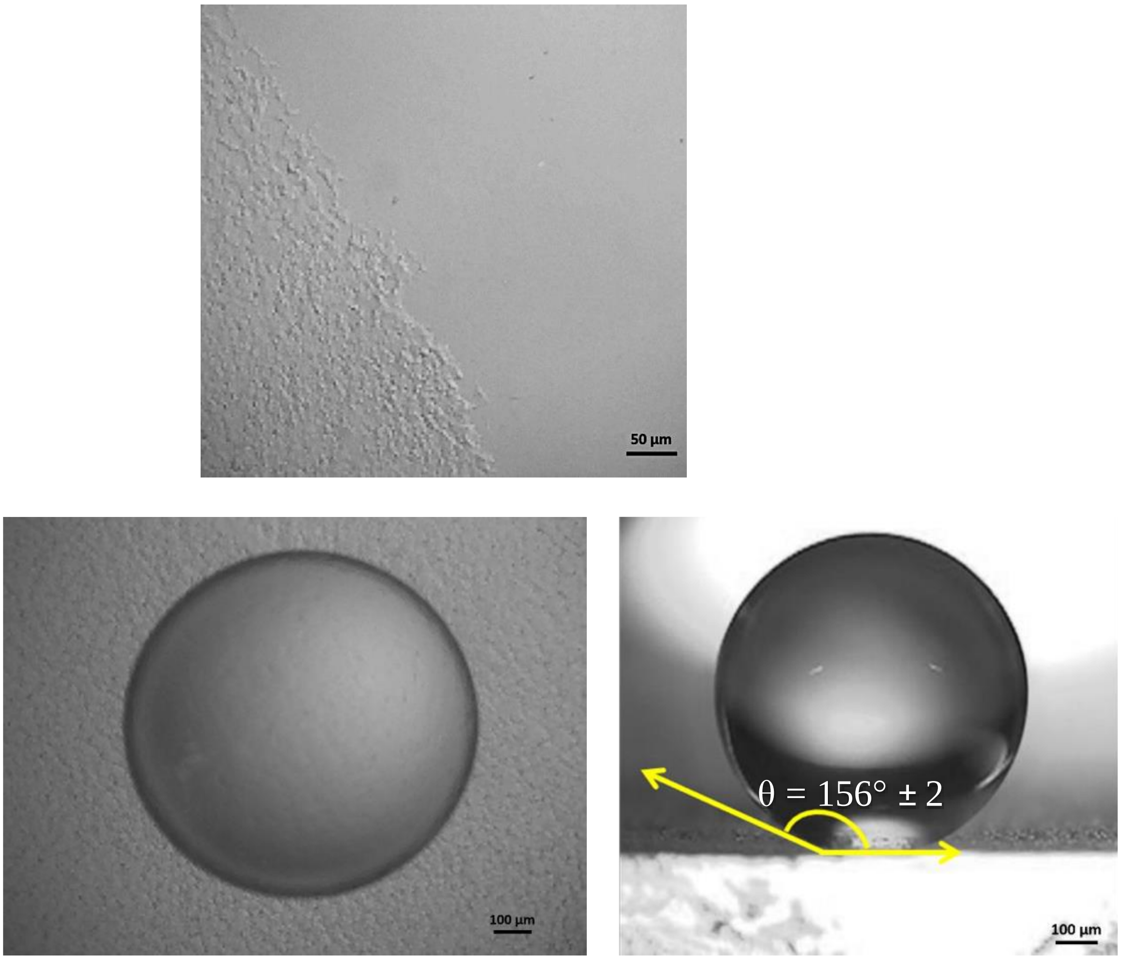

Figure S1. Nanostructured superhydrophobic surface.

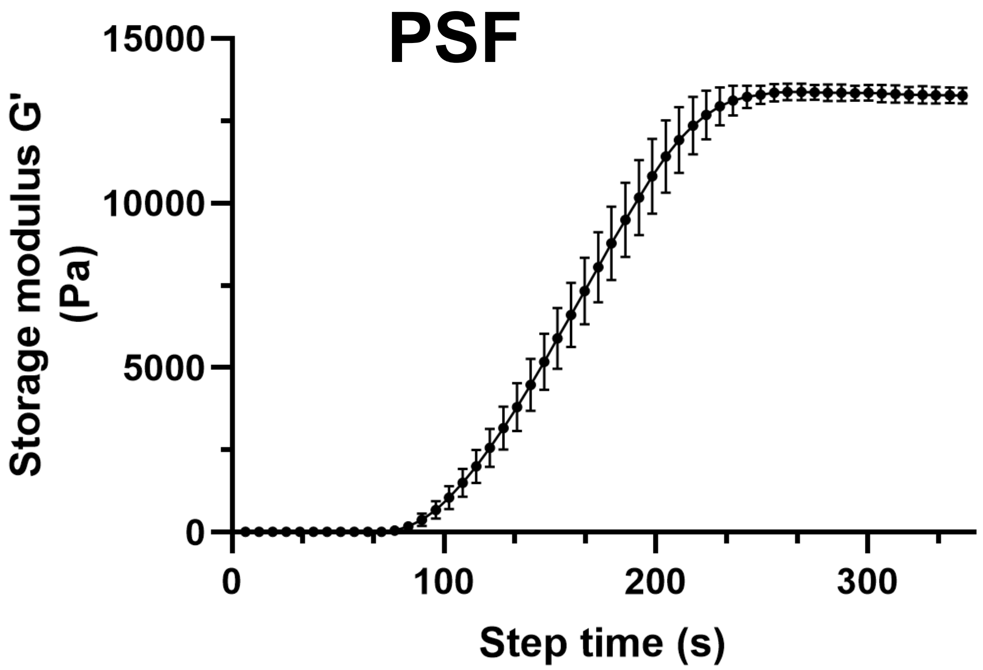

Figure S2. Rheological analysis of PSF hydrogel. Rheologic measurements of shear storage modulus (G′) for PSF hydrogel 9% PEGDa w/v. [48].

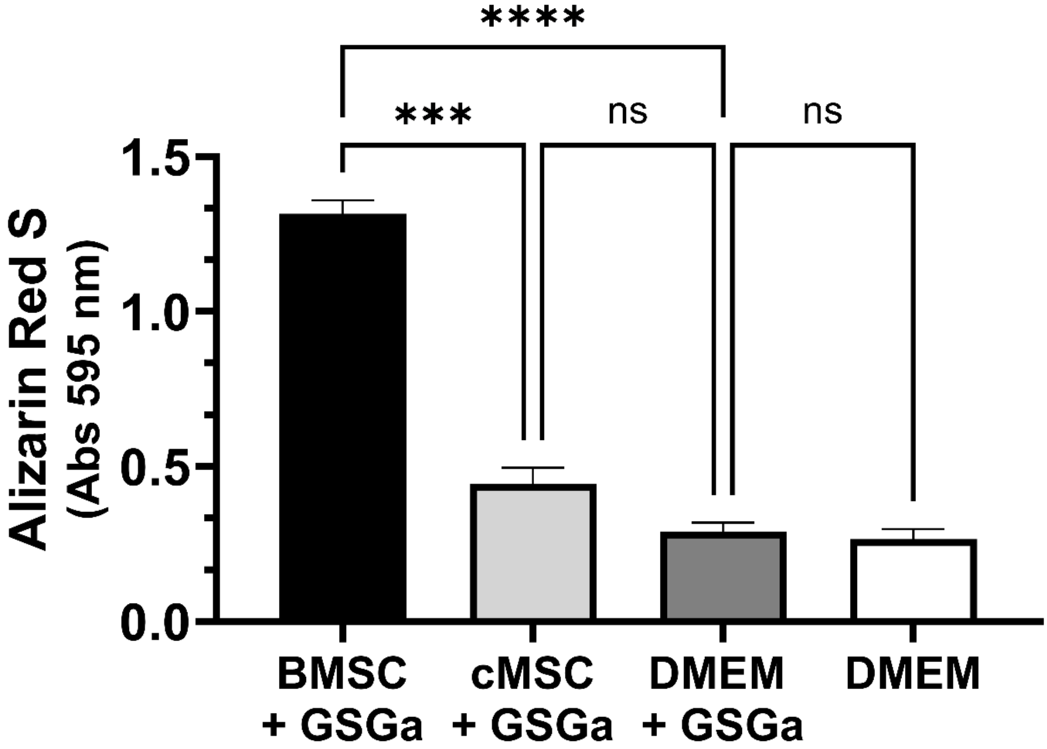

Figure S3. Quantitative analysis of the Alizarin Red staining of hMSCs after GSGa treatment. 2D cell cultures of hMSCs seeded at cell density of 5 × 10^3 cells/cm^2 and cultured for three days in the presence and in the absence of GSGa (680 µg/mL). Alizarin Red S staining was performed on the cell culture medium after centrifugation for evaluating the presences of calcium deposits.

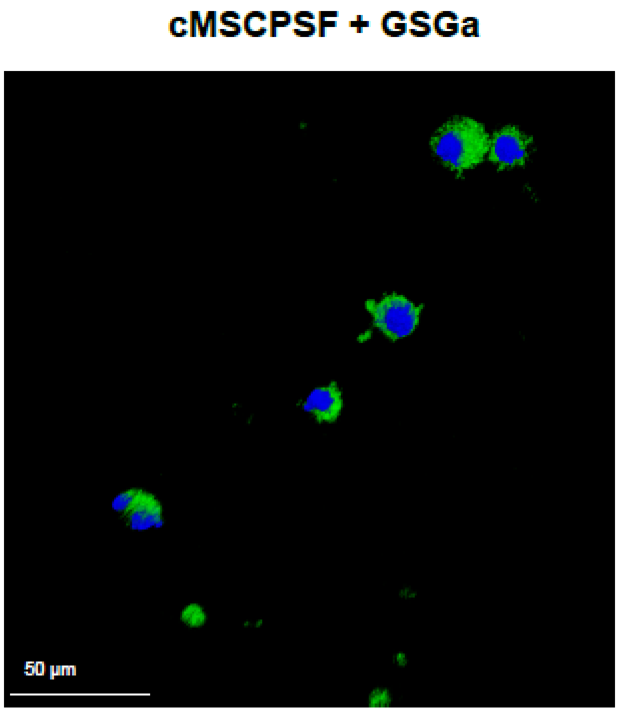

Figure S4. Osteocalcin expression in 3D MSCPSF grown in the presence of GSGa. 3D confocal micrograph of cMSCPSF+GSGa; the osteocalcin expression was assessed by Ab-osteocalcin- Alexa-Fluor 488 nm (in green), the nuclei were stained with Hoechst 33,342 (in blue); Scale bars are of 50 µm.

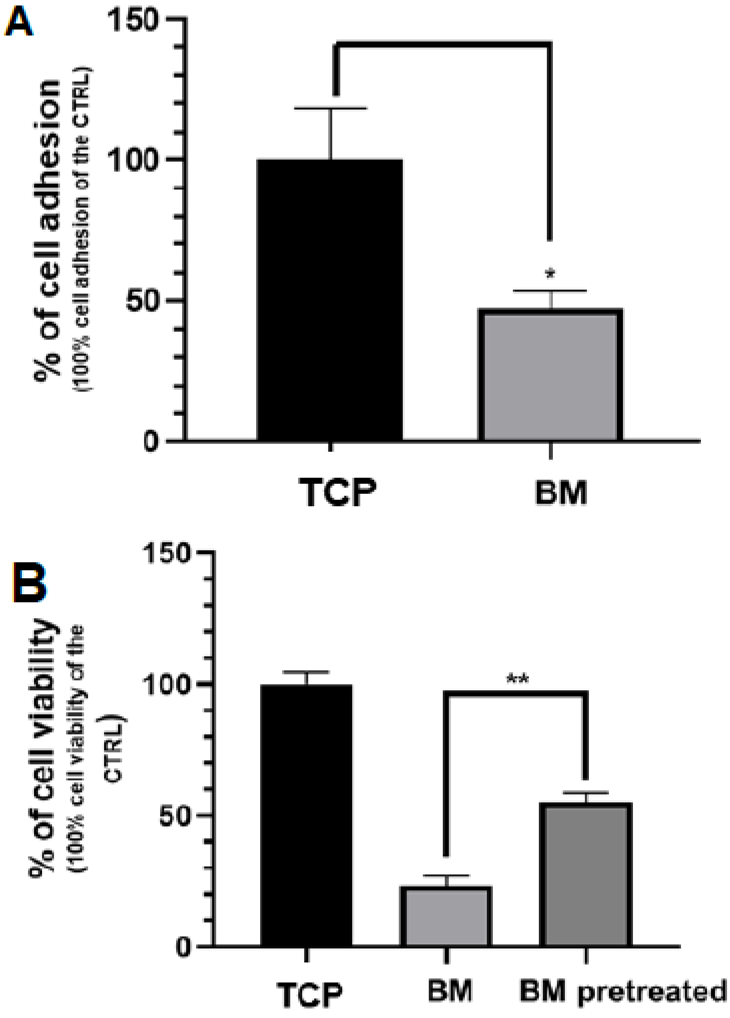

Figure S5. Cell adhesion and viability on Bone matrix. A) cell adhesion assay of MDA cells after 2 h from cell seeding on BM; 100% was the control represented by MDA-MB-231 cells adhered to the tissue culture plate (TCP); B) cell viability WST-1 assay of MDA-MB-231 cells after 24 h of cell growth with BM and pretreated BM compared to control (TCP). The data were obtained by three independent experiments. Error bar indicates S.D. * p value ≤ 0.05, ** p value ≤ 0.01.

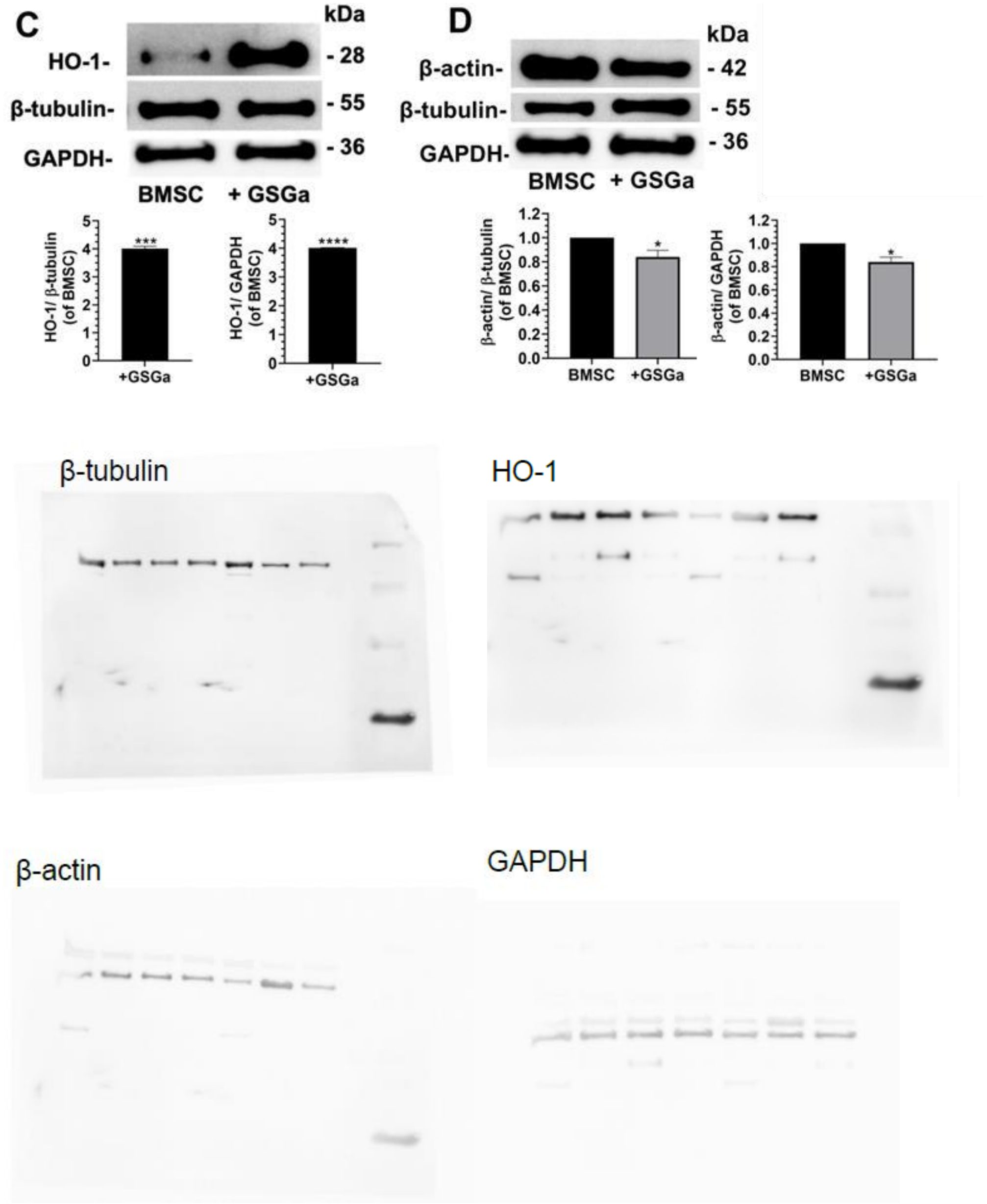

Figure S6. Original Western Blotting of Figure 1.

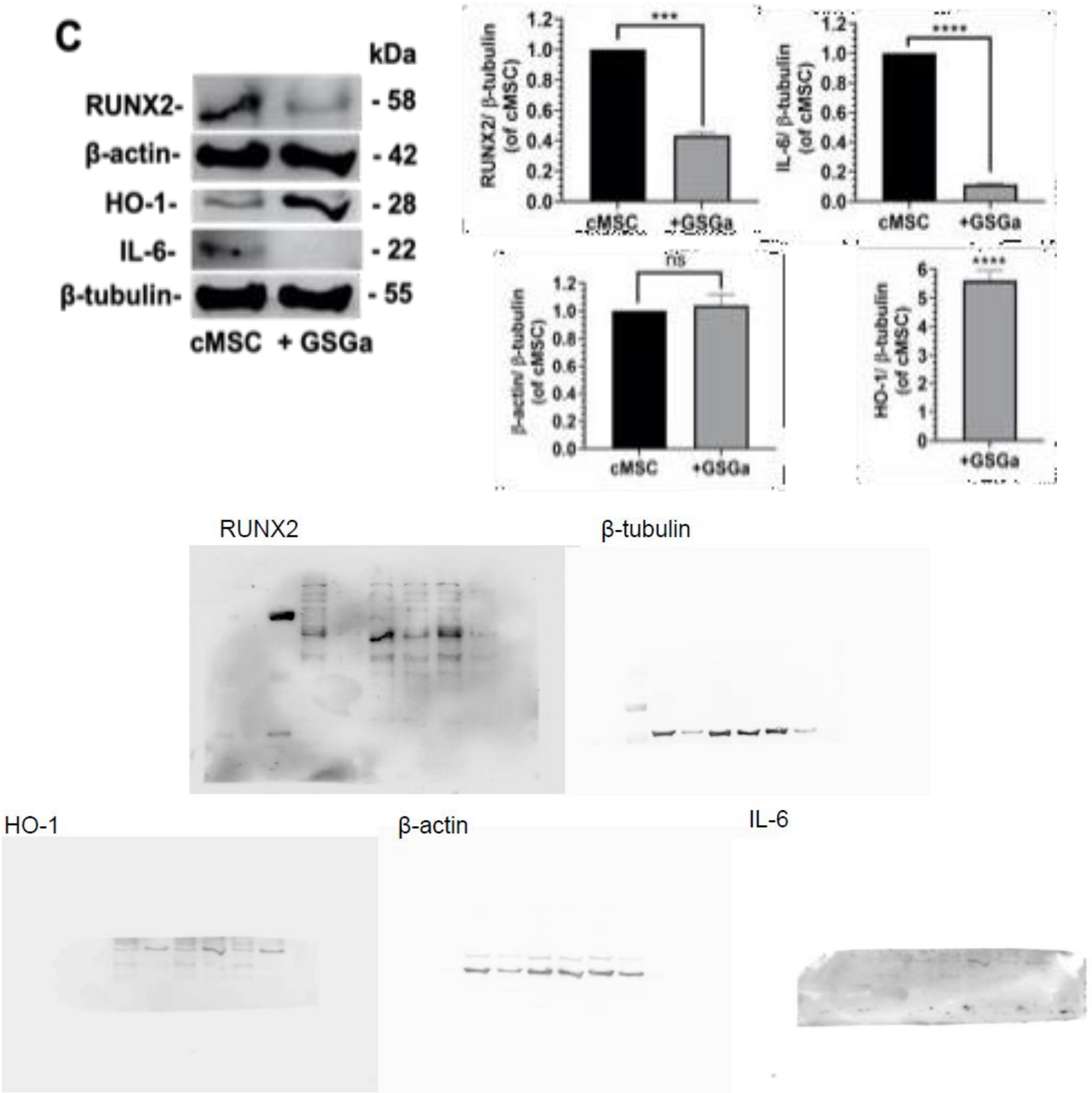

Figure S7. Original Western Blotting of Figure 2.

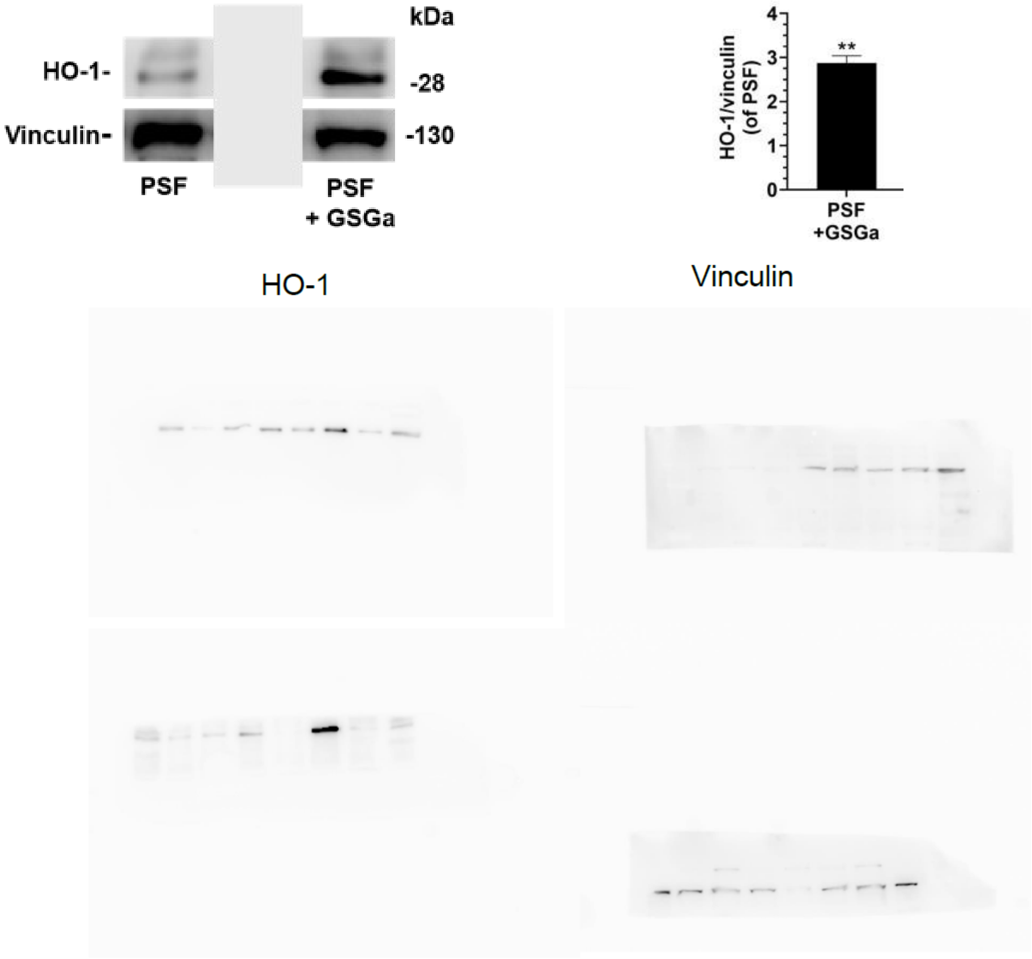

Figure S8. Original Western Blotting of Figure 3.

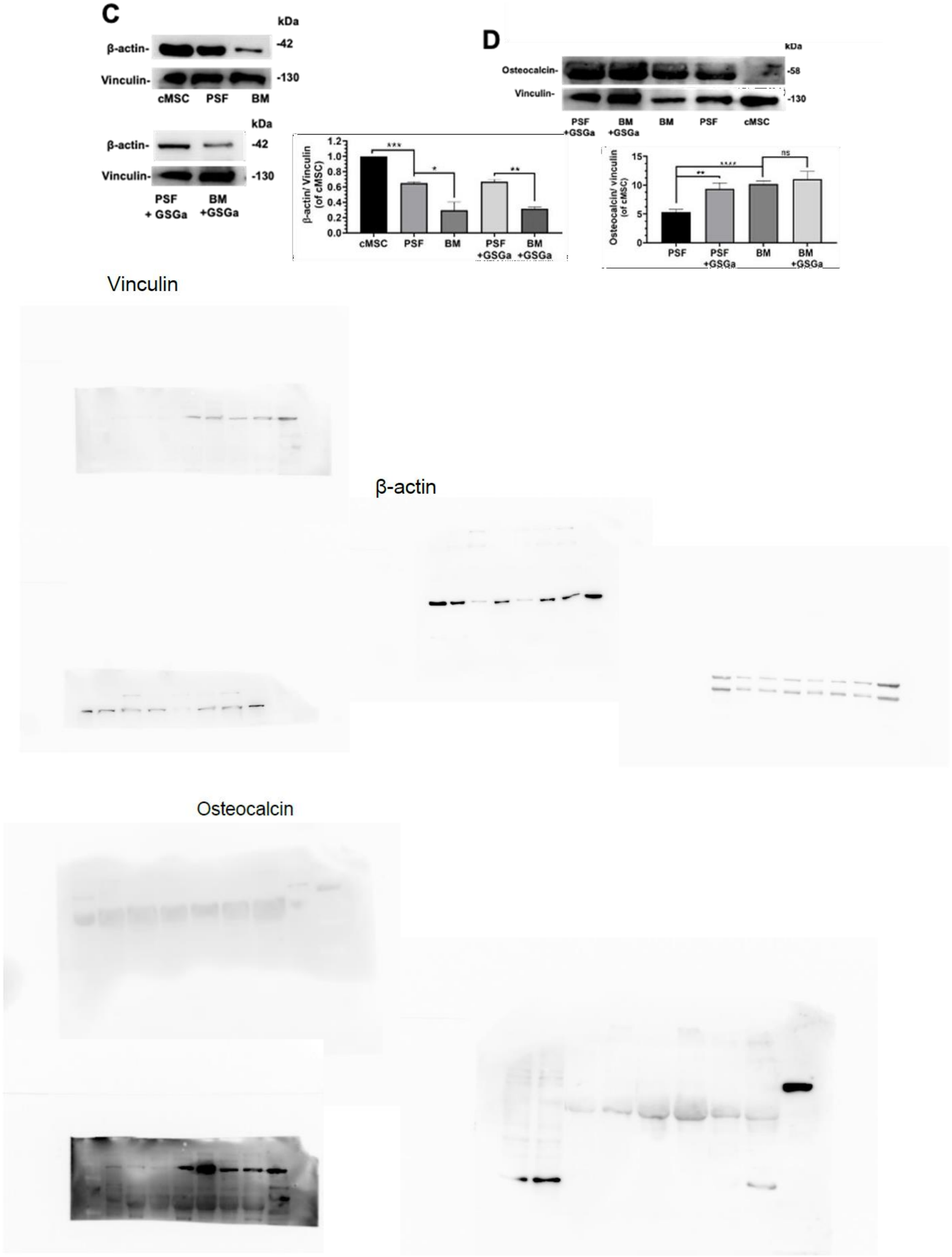

Figure S9. Original Western Blotting of Figure 6.
